# OVSCORE - a validated score to identify ovarian cancer patients not suitable for primary surgery

**DOI:** 10.3892/ol.2014.2630

**Published:** 2014-10-24

**Authors:** JULIA DORN, HOLGER BRONGER, RONALD KATES, JULIA SLOTTA-HUSPENINA, BARBARA SCHMALFELDT, MARION KIECHLE, ELEFTHERIOS P. DIAMANDIS, ANTONINUS SOOSAIPILLAI, MANFRED SCHMITT, NADIA HARBECK

**Affiliations:** 1 Department of Obstetrics and Gynecology, Klinikum rechts der Isar, Technical University of Munich, Munich, Germany; 2 Department of Pathology, Klinikum rechts der Isar, Technical University of Munich, Munich, Germany; 3 Department of Pathology and Laboratory Medicine, Mount Sinai Hospital, Department of Laboratory Medicine and Pathobiology, University of Toronto, Toronto, ON, Canada; 4 Department of Obstetrics and Gynecology, Breast Center, Klinikum Grosshadern, University of Munich, Munich, Germany

**Keywords:** kallikrein-related peptidases, kallikrein, ovarian cancer, surgical success, residual tumor, prognosis

## Abstract

Following primary debulking surgery, the presence of a residual tumor mass is one of the most important prognostic factors in ovarian cancer. In a previous study, we established the OVSCORE, an algorithm to predict surgical outcome, based on the clinical factors of nuclear grading and ascitic fluid volume, plus the cancer biomarkers, kallikrein-related peptidases (KLKs), KLK6 and KLK13. In the present study, OVSCORE performance was tested in an independent ovarian cancer patient cohort consisting of 87 patients. The impact of KLKs, KLK5, 6, 7 and 13 and other clinical factors on patient prognosis and outcome was also evaluated. The OVSCORE proved to be a strong and statistically significant predictor of surgical success in terms of area under the receiver operating characteristic curve (ROC AUC, 0.777), as well as positive and negative predictive value in this independent study group. KLK6 and 13 individually did not show clinical relevance in this cohort, but two other KLKs, KLK5 and KLK7, were associated with advanced FIGO stage, higher nuclear grade and positive lymph node status. In the multivariate Cox regression analysis for overall survival (OS), KLK7 had a protective impact on OS. This study confirms the role of KLKs in ovarian cancer for surgical success and survival, and validates the novel OVSCORE algorithm in an independent collective. As a key clinical application, the OVSCORE could aid gynecological oncologists in identifying those ovarian cancer patients unlikely to benefit from radical surgery who could be candidates for alternative therapeutic approaches.

## Introduction

The prognosis of patients with ovarian cancer is generally poor owing to an advanced FIGO stage at diagnosis and often inefficient primary debulking surgery, the most important prognostic factors in ovarian cancer (1). The presence of a residual tumor is essentially the only factor that can be affected by the gynecological oncologist (2).

In ovarian cancer, cutting-edge therapy consists of radical debulking surgery that aims for complete tumor resection, followed by chemotherapy. Despite advancements in surgical techniques, complete debulking (no macroscopically evident residual tumor mass) is only achieved in ~30% of ovarian carcinoma patients, and radical tumor resection may cause considerable morbidity (3). High morbidity is certainly acceptable if complete debulking with its attendant survival advantage can be achieved, but it must be viewed critically for patients left with a residual intra-abdominal tumor and a resulting shorter life span (4).

Such patients would be ideal candidates for alternative therapeutic approaches, such as neoadjuvant chemotherapy with or without subsequent surgery. However, identification of these patients is difficult, as no standardized selection criteria have been put forward so far. Neither radiological imaging nor a variety of analyzed protein and gene expression parameters have been shown to predict surgical success accurately (5–7).

As reported previously (8), in order to predict surgical success, we have developed the OVSCORE algorithm on the basis of a logistic regression model involving two clinical factors and two tumor biological factors, namely ascitic fluid volume and nuclear grading, and kallikrein-related peptidases (KLKs), KLK6 and KLK13, which are members of a serine protease family and are known to be associated with ovarian cancer progression and metastasis (9,10). In our previous trial, employing the OVSCORE, the presence of a residual tumor mass following radical surgery could be estimated, with good in-sample predictive performance [area under the receiver operating characteristic curve (ROC AUC), 0.833] (8).

Other than being markers in the OVSCORE algorithm to predict surgical success, the majority of members of the KLK-family have been reported to be significant ovarian cancer biomarkers (9,10). For example, high KLK5 and KLK6 tumor tissue levels are associated with an advanced disease stage and significantly shorter progression-free survival (PFS) and overall survival (OS) times (11–14). Recently, we found that ovarian cancer patients with high KLK7 tumor tissue ELISA-levels had a two-fold lower risk of mortality or relapse compared with patients who displayed low levels (15). Finally, KLK13 expression in ovarian cancer tumor tissue has been shown to correlate with early-stage disease and favorable OS (16). These properties motivated the evaluation of the clinical impact of the biomarkers KLK5–7 and KLK13 in the present study.

The present study reports a retrospective validation of OVSCORE in an independent data set comprised of 87 patients with ovarian cancer, focusing in particular on its accuracy in predicting the presence of a residual tumor following primary surgery. Furthermore, the prognostic impact of established clinical factors and the analyzed KLK5–7 and KLK13 concentrations in tumor tissue was also analyzed in this independent collective.

## Patients and methods

### Patients

A total of 87 patients with ovarian cancer of International Federation of Gynecology and Obstetrics (FIGO) stage I–IV who were treated between 1995 and 2008 at the Department of Obstetrics and Gynecology, Klinikum rechts der Isar, Technical University of Munich (Munich, Germany), were enrolled in the present retrospective biomarker study. Standard surgical procedures were performed including pelvic and para-aortic lymphadenectomy and if indicated, partial resection of the small and large intestine, peritonectomy and upper abdominal surgery. All patients provided written informed consent and the study was approved by the ethics committee of Klinikum rechts der Isar, Technical University of Munich. Following surgery, all patients received adjuvant treatment, including platinum-based chemotherapy (carboplatin AUC5 and Paclitaxel, 175 mg/sqm, body surface area, every three weeks), according to consensus recommendations at that time. The median age of the patients at the time of surgery was 58 years (range, 25–83 years). The median time of follow-up was 50 months (range, 1–166) for OS and 28 months (range, 1–159) for PFS. The clinical and histomorphological factors are presented in Table I. In total, 69 tumors (79.3%) were of the serous histotype, 9 (10.3%) were endometrioid, 4 (4.6%) were mucinous and 5 (5.7%) belonged to other histotypes.

### Preparation of ovarian cancer tumor tissue extracts

Extracts from ovarian cancer primary tumor tissues were prepared as described previously (17). Tumor tissue samples were collected during surgery, inspected and classified by a pathologist, and then stored in liquid nitrogen. For extraction, deep-frozen specimens of 200–500 mg weight were pulverized and then resuspended in Tris-buffered saline [TBS; 0.02 M Tris-HCl and 0.125 M NaCl (pH 8.5)] containing 1% (w/v) of the non-ionic detergent Triton X-100 (Sigma Aldrich, Munich, Germany). Subsequent to extraction and ultracentrifugation at 100,000 × g for 45 min, the supernatant was collected, aliquoted and stored in liquid nitrogen until further use.

### ELISA tests for the quantitation of KLK5–7 and KLK13 in ovarian cancer tumor tissue extracts

KLK5–7 and KLK13 antigen concentrations were determined in ovarian cancer tumor tissue extracts by non-commercial in-house ELISA test formats (18). For this purpose, monoclonal capture antibodies and detection antibodies to KLK5–7 and KLK13 proteins were generated by immunizing mice with recombinant human KLK5–7 and KLK13. Lower detection limits of the various KLK ELISAs were 0.05 ng/ml for KLK5, 6 and 13, and 0.2 ng/ml for KLK7. In these ELISA formats, no cross-reactivities with any other member of the human KLK family were detected. KLK antigen values were expressed as ng analyte/mg protein, which was determined in the tissue extracts by the Pierce BCA method (8).

### Statistical analyses

All outcome and explanatory variables were coded in a manner consistent with our previous study (8). Outcome variables were PFS, OS and residual tumor presence (RT); RT was defined as 1 if a macroscopic residual tumor mass was visible and zero if completely absent.

Ascites volume, age, nuclear grade and nodal status were coded as binary variables: Ascites, >500 vs. ≤500 ml; age, >60 vs. ≤60 years; nuclear grade, G3 vs. G1/G2; and nodal status, 0 for N0, otherwise 1. FIGO status was coded by three binary indicators: Stage II/III/IV vs. I; stage III/IV vs. I/II; and stage IV vs. I/II/III.

For validation of the OVSCORE, ‘virtual’ fractional ranks of KLK6 and KLK13 were coded with respect to the distributions of antigens in the original data set (8), using interpolation as required.

The OVSCORE was then computed for each patient by multiplying the coded values of the factors by the logarithm of the corresponding odds coefficient (8). The procedure can be summarized by the following formula: OVSCORE = 2.57 × (A) + 1.07 × (G) + 2.14 × (rKLK6) − 2.00 × (rKLK13) − 5.41, where ‘A’ is defined as one if ascites >500 ml and zero otherwise, ‘G’ is one if grade is G3 and zero otherwise, ‘rKLK6’ is the virtual fractional rank of KLK6 and ‘rKLK13’ is the virtual fractional rank of KLK6. The fractional rank coding scheme for validation is intended to ensure that equal values of OVSCORE in the two independent cohorts correspond as closely as possible to the same tumor biological interpretation, even if the disease stage distributions in the two cohorts differ substantially.

The OVSCORE was used to construct a ROC for prediction of surgical success (RT) in the current validation sample. The ROC AUC was calculated as an out-of-sample performance indicator for validation. The out-of-sample positive predictive value (PPV) and negative predictive value (NPV) are also reported for the median and scaled cutoffs; the scaled cutoff is defined for this statistic as the fractional rank of OVSCORE equal to the percentage of patients with an RT of zero.

Spearman correlations between continuous variables and Mann-Whitney or Kruskal-Wallis tests for associations between continuous and categorical variables were computed. Impacts on PFS and OS were estimated by Cox’s proportional hazards regression model using forward selection, and then expressed as hazard ratios (HR) with respect to the defined coding.

Analyses were performed using SPSS software (SPSS, Inc., Chicago, IL, USA). P<0.05 was used to indicate a statistically significant difference.

## Results

### Validation of the OVSCORE algorithm to predict surgical success in ovarian cancer patients

**Figure 1:**
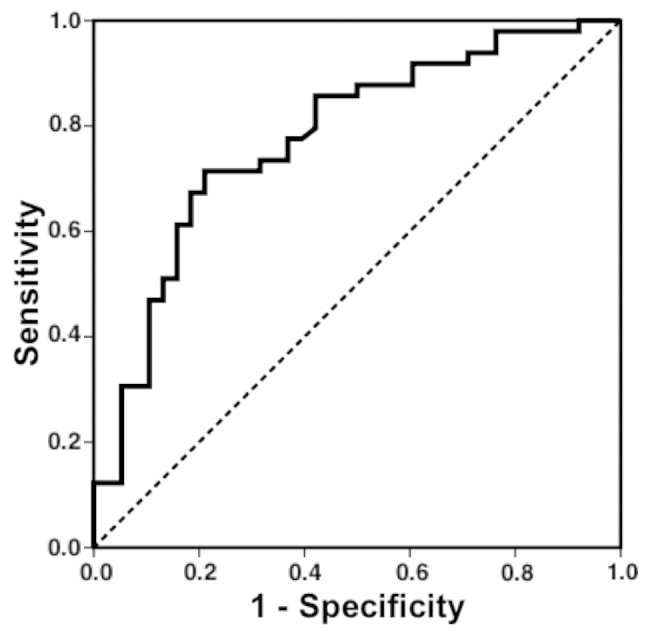
ROC curve for prediction of residual tumor mass status by employing the OVSCORE. The area under the curve is 0.777.

Based on our previous study (8), the OVSCORE algorithm was developed to predict surgical success in ovarian cancer patients undergoing intra-abdominal debulking surgery in order to reduce the tumor size. The score, encompassing the clinical factors of ascitic fluid volume and nuclear grading, plus the cancer biomarkers KLK6 and KLK13, is designed to predict the efficiency of the intra-abdominal debulking procedure. In the present study, the predictive performance of the OVSCORE algorithm was tested in an independent set of 87 primary ovarian cancer patients. The predictive performance was comparable to that obtained in the original cohort of 142 ovarian cancer patients: AUC ROC for pilot study, 0.833; AUC ROC this study, 0.777 (Fig. 1). The corresponding out-of-sample quality measures for prediction of RT in terms of sensitivity, specificity, PPV and NPV for median and scaled cutoffs are summarized in Table II. Using the median cutoff, for example, one can expect that an unfavorable (positive) OVSCORE test would be correct in predicting a residual tumor in ~81% of cases; a favorable (negative) OVSCORE test would be correct in predicting surgical success in ~68% of cases.

### Correlations

**Figure 2:**
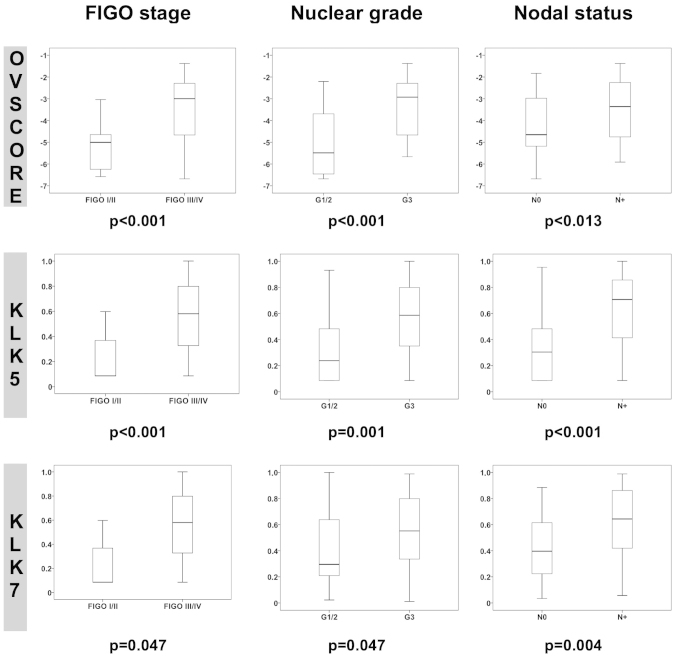
Boxplot diagrams showing correlations between FIGO stage, nuclear grade and nodal status with KLK5^a^, KLK7^a^ and OVSCORE^b^ in ovarian cancer patients. KLK5, KLK7 and OVSCORE values are significantly associated with advanced FIGO stage, higher nuclear grade and positive lymph node status. FIGO, International Federation of Gynecology and Obstetrics; KLK, kallikrein-related peptidase.

Significant Spearman’s rank correlations (R_s_) were found between KLK5 and KLK7 (R_s_, ~0.6) and between KLK7 and KLK13 (R_s_, ~0.3). Associations of the clinically established variables of FIGO stage, nuclear grade, nodal status and ascitic fluid volume, with cancer biomarkers KLK5–7 KLK13, serum biomarker cancer antigen (CA)-125 and the OVSCORE were quantified employing the Mann-Whitney U test (Fig. 2): KLK5, KLK7 and the OVSCORE (as well as CA-125 in serum, data not shown) were associated with advanced FIGO stage (FIGO III/IV), higher nuclear grade (G3) and positive nodal status (N^+^); KLK13 was associated with nodal status (data not shown), while KLK6 was not significantly associated with these clinical factors.

### Assessment of prognostic impact of clinical factors, CA-125, KLK5–7 and KLK13

The impact of FIGO stage, nuclear grading, nodal status, ascitic fluid volume, residual tumor mass, CA-125, KLK5–7 and KLK13 on OS and PFS, as assessed by uni- and multivariable Cox analysis, are summarized in Table III. The clinical factors were significant univariate predictors of OS and PFS. The OVSCORE itself was significantly associated with OS and PFS in univariate analysis; KLK5–7 and KLK13 were not individually significant for either OS or PFS, whereas CA-125 was not significant for OS or PFS, the P value for OS was 0.06.

The multivariate Cox model for OS contained residual tumor mass, nuclear grade and KLK7 as independent factors. Adjusted for residual tumor mass and nuclear grade, KLK7 entered the model as a protective marker (HR, 0.41; Table IV). Residual tumor mass was the only independent marker in the model for PFS.

## Discussion

For ovarian cancer, the major traditional prognostic factors are FIGO stage at the time of diagnosis and size of residual tumor mass following cytoreductive surgery (1). Other established clinical prognostic factors are age, performance status, type of histology, nuclear grade and presence/amount of peritoneal ascitic fluid. However, owing to the lack of suitable biomarkers, at present, ovarian cancer management does not use any prognostic or therapy response predicting factors, with regard to the course of the disease or a patient’s risk to develop disease recurrence.

In our previous study, the OVSCORE algorithm was developed to predict the surgical outcome in the primary debulking surgery of ovarian cancer patients (8). This score encompasses the clinically relevant factors of ascitic fluid volume and nuclear grading, plus two novel cancer biomarkers, the serine proteases KLK6 and KLK13.

Broad scientific interest has been focused on the KLKs, since the majority of the fifteen KLK family members are believed to contribute to ovarian cancer progression and metastasis (9,10). Four KLKs (KLK4–6 and KLK15) are linked to the poor prognosis of ovarian cancer patients, while higher KLK9 and KLK14 levels are associated with a favorable course of the disease. For certain KLKs (KLK7, 8, 10, 11 and KLK13), the clinical relevance is not yet clear, since, depending on the method of detection (mRNA or protein, ELISA or IHC) and/or FIGO stage (early or advanced), these KLKs can be associated with either a poorer or more favorable disease course.

The present study validated the clinical relevance of the multiparametric OVSCORE to predict surgical success in an independent set of ovarian cancer patients. The performance of the score in this set was found to be comparable to that in the original collective of 142 ovarian cancer patients of the pilot study (ROC AUC pilot study, 0.833; AUC ROC this study, 0.777), even though complete debulking rates as a whole were higher in the previous study.

The OVSCORE could easily be calculated prior to definitive surgery by estimating the pre-operative ascitic fluid volume by ultrasound and analyzing tumor biopsies obtained by laparoscopy or CT-guided for nuclear grading and KLK tissue analysis. Applying the presurgical OVSCORE, ovarian cancer patients for whom complete tumor resection would be difficult to achieve could be identified. Such patients could be candidates for alternative clinical approaches, such as neoadjuvant or exclusive chemotherapy.

Furthermore, the OVSCORE could also support pre-operative risk stratification: e.g., in ovarian cancer patients with substantial co-morbidity, a favorable OVSCORE would reinforce the decision for radical surgery, whereas an unfavorable OVSCORE would suggest considering alternative therapeutic approaches, for instance pre-operative chemotherapy.

Apart from validating the clinical utility of the OVSCORE, the present study evaluated the clinical impact of the various single parameters accounting for the OVSCORE algorithm. Large ascitic fluid volumes and higher nuclear grading are established clinical prognostic factors known to be associated with higher stage and inferior survival rates (1,8). As aforementioned, KLK6 is known to be an unfavorable prognostic ovarian cancer biomarker (13,14), while KLK13 expression is correlated with early-stage disease and favorable OS (16). In view of recent studies showing that elevated KLK5 and KLK7 values are associated with advanced stage, higher nuclear grade and a poor prognosis in ovarian cancer patients (11,12,19–21), the clinical impact of these two additional KLKs was tested.

In Cox’s univariate analysis, the prognostic impact on OS and PFS by the established clinical factors of FIGO stage, residual tumor volume subsequent to surgery, nuclear grade and ascitic fluid volume, as well as the OVSCORE, could be confirmed, but none of the KLKs alone or CA-125 in serum showed significance.

In multivariate Cox analysis, only residual tumor mass and, for OS, nuclear grade and KLK7 were of clinical significance. Notably, in this multivariate context, KLK7 was found to be a protective marker (HR, 0.41). This result is consistent with our previous findings in an independent ovarian cancer patient cohort demonstrating that higher KLK7 protein levels, as assessed by ELISA, are associated with an improved patient outcome (15). However, there are conflicting studies with regard to KLK7, which state that it may be associated with a worse prognosis and a poor response to chemotherapy (8,19,20,22–25). However, contradictory findings have already been reported for KLK7 in breast cancer. Holzscheiter *et al* (26) found high full-length KLK7 mRNA levels in breast cancer tissue to be a favorable prognostic marker, while Talieri *et al* (27) reported KLK7 expression to be associated with shorter survival. A possible explanation for this discrepancy may be the fact that in contrast to the cohort of Holzscheiter *et al* (26), most of the patients analyzed by Talieri *et al* (27) were subjected to adjuvant therapy.

The present ovarian cancer cohort was uniformly treated by adjuvant platinum-containing polychemotherapy (except if not indicated or not suitable). By contrast, only 30% of the patients included in the study by Kyriakopoulou *et al* (20) were treated with carboplatin and only 16% with paclitaxel. Hence, a therapeutic effect analogous to that hypothesized by Holzscheiter *et al* (26) is possible. Furthermore, in the patient cohort analyzed by Shan *et al* (19), more patients were of earlier stage, with 30% FIGO stage I/II compared with 17% FIGO stage I/II in the present cohort, and fewer succumbed; 50% compared with 68% in the present patient group.

Taken these findings as a whole, KLKs have recently emerged as novel, promising predictive factors in ovarian cancer. In particular, the present study has confirmed earlier findings showing that KLK5 is associated with advanced and more aggressive disease, and that KLK7 may be a favorable prognostic marker in ovarian cancer. The key clinical finding of this study is the validation of the previously developed OVSCORE in an independent patient cohort. The OVSCORE could aid in the identification of patients who do not benefit from currently recommended therapeutic regimens.

## Figures and Tables

**Table I:** Patient characteristics.

Clinicopathological parameters	n (%)
FIGO stage	
I	11 (12.6)
II	4 (4.6)
III	50 (57.5)
IV	22 (25.3)
Lymph node status	
N0	27 (31.1)
N^+^	41 (47.1)
Not known	19 (21.8)
Residual tumor, mm	
0	38 (43.7)
>0	49 (56.3)
Ascitic fluid volume, ml	
None	20 (23.0)
≤500	22 (25.3)
>500	45 (51.7)
Nuclear grading	
G1	3 (3.4)
G2	17 (19.5)
G3	67 (77.0)
Response to chemotherapy	
Progress	12 (13.8)
No change	3 (3.4)
Complete remission	25 (28.7)
Partial remission	5 (5.7)
Not known	42 (48.3)
Deceased	
No	25 (28.7)
Yes	59 (67.8)
Not known	3 (3.4)
Relapsed	
No	17 (19.5)
Yes	62 (71.3)
Not known	8 (9.2)

FIGO, International Federation of Gynecology and Obstetrics.

**Table II:** Out-of-sample quality measures for prediction of residual tumor presence.

OVSCORE	Sensitivity	Specificity	PPV	NPV
OVSCORE with median cutoff, %	71.4	78.9	81.4	68.20
OVSCORE with scaled cutoff, %	73.5	65.8	73.5	65.80

a OVSCORE encompasses ascitic fluid volume, nuclear grading, KLK6 and KLK13.

PPV, positive predictive value; NPV, negative predictive value; KLK, kallikrein-related peptidase.

**Table III:** Univariate Cox regression analysis to determine the association of clinical factors and cancer biomarkers with ovarian cancer patient survival (n=84).

	Overall survival			Progression-free survival		
		
Variable	HR	95% CI	P-value	HR	95% CI	P-value
FIGO stage	3.5	1.4–8.9	0.007	2.8	1.2–6.6	0.019
Nuclear grade	3.2	1.5–6.7	0.003	2.6	1.3–5.0	0.007
Residual tumor mass, mm	34.4	11.2–105.9	<0.001	1.1	1.0–1.1	<0.001
Residual tumor mass, mm (0 vs. >0)	4.5	2.5–8.1	<0.001	3.3	1.9–5.8	<0.001
Ascitic fluid volume, ml	2.3	1.3–3.9	0.002	2.2	1.3–3.7	0.003
OVSCORE^a^	1.4	1.2–1.7	<0.001	1.3	1.1–1.6	0.001
CA-125	1.4	1.0–1.9	0.060			0.100

a OVSCORE encompasses ascitic fluid volume, nuclear grading, KLK6 and KLK13.

HR, hazard ratio; CI, confidence interval; FIGO, International Federation of Gynecology and Obstetrics; KLK, kallikrein-related peptidase; CA-125, cancer antigen 125.

**Table IV:** Multivariable Cox regression analysis to determine the association of clinical factors and cancer biomarkers with ovarian cancer patient survival (n=84).

	Overall survival			Progression-free survival		
		
Variable	HR	95% CI	P-value	HR	95% CI	P-value
Residual tumor mass, mm (0 vs >0)	4.6	2.5–8.6	<0.001	3.31	1.90–5.77	<0.001
Nuclear grade	2.6	1.2–5.8	0.016	-	-	NS
KLK7	0.4	0.2–1.0	0.041	-	-	NS

NS, not significant; HR, hazard ratio; CI, confidence interval; FIGO, International Federation of Gynecology and Obstetrics; KLK, kallikrein-related peptidase.
